# Synthesis, crystal structure determination, Hirshfeld surface and crystal void analyses, inter­action energy calculations and energy frameworks of *N*-(2-methyl­phen­yl)-*N*′-pivaloyl­thio­urea

**DOI:** 10.1107/S205698902600890X

**Published:** 2026-09-03

**Authors:** Sharatha Kumar, Tuncer Hökelek

**Affiliations:** a Department of Chemistry Yenepoya Institute of Arts Science Commerce and Management Mangaluru Yenepoya University (Deemed to be university) 575013 Karnataka India; b Hacettepe University, Department of Physics, 06800 Beytepe-Ankara, Türkiye; Vienna University of Technology, Austria

**Keywords:** crystal structure, pivalo­yl, thio­urea-derivative, 2-methyl­phenyl substituent, inter­molecular inter­actions

## Abstract

The title compound [systematic name: 3-(2,2-di­methyl­propano­yl)-1-(2-methyl­phen­yl)thio­urea], C_13_H_18_N_2_OS, consists of methyl­phenyl and pivaloyl moieties attached to the N atoms of thio­urea. The latter is twisted by 78.08 (10)° with respect to the phenyl ring. An intra­molecular N—H⋯O hydrogen bond with an *S*(6) ring motif consolidates the mol­ecular conformation. In the crystal, C—H⋯S hydrogen bonds link two mol­ecules, enclosing *R*^2^_2_(14) ring motifs, into centrosymmetric dimers. Furthermore, π–π stacking and C—H⋯π(ring) inter­actions are present. Hirshfeld surface analysis revealed that the most important contributions to the crystal packing are from H⋯H (62.4%), H⋯S/S⋯H (13.6%) and H⋯C/C⋯H (12.9%) inter­actions. The volume of the crystal voids and the percentage of free space were calculated to be 91.57 Å^3^ and 13.35%, showing that there is no large cavity in the crystal packing. A C—H⋯S hydrogen-bonding energy of −5.4 kJ mol^−1^ was calculated. Evaluation of the electrostatic, dispersion and total energy frameworks indicates that the packing is dominated by dispersion energy contributions.

## Abstract 2

The asymmetric unit of the title compound comprises an ordered mol­ecule, consisting of methyl­phenyl and pivaloyl moieties attached to the N atoms of the central thio­urea entity.

## 1. Chemical context

N-substituted *N*-acyl­thio­ureas are important synthetic inter­mediates for the preparation of a variety of heterocyclic compounds through cyclization reactions (Saeed *et al.*, 2016 ▸; Aly *et al.*, 2016 ▸). They have also attracted considerable inter­est as precursors for anion receptors (Blažek Bregovic *et al.*, 2015 ▸; Zhang *et al.*, 2006 ▸), organocatalysts (Guang *et al.*, 2015 ▸; Mao *et al.*, 2013 ▸; Saeed *et al.*, 2017 ▸), corrosion inhibitors (Al-Abbassi *et al.*, 2023 ▸), and non-ionic surfactants (Ullah *et al.*, 2015 ▸). Moreover, numerous acyl­thio­urea derivatives exhibit a broad spectrum of biological activities, including anti­convulsant (Bielenica *et al.*, 2016 ▸), anti­cancer (Ruswantor *et al.*, 2015 ▸; Kumar & Chimni, 2015 ▸; Ribeiro *et al.*, 2023 ▸), anti­diabetic (Faidullah *et al.*, 2011 ▸), anti-inflammatory (Canatar *et al.*, 2023 ▸; Mohamed *et al.*, 2021 ▸), anti-HIV (Singh & Ganguly, 2018 ▸), anti­microbial (Zhong *et al.*, 2008 ▸; Aher *et al.*, 2009 ▸), urease inhibitory (Khan *et al.*, 2014 ▸), herbicidial (Xu *et al.*, 2003*a* ▸), and insecticidal (Xu *et al.*, 2003*b* ▸) activities. Thio­urea derivatives containing sulfur- and nitro­gen-donor atoms have also attracted considerable attention in medicinal chemistry owing to their inter­esting pharmacological properties. In particular, thio­urea-based compounds have been investigated as aromatase inhibitors for the treatment of estrogen-dependent breast cancer (Pingaew *et al.*, 2018 ▸). Their anti­cancer activity has also been attributed to inter­ference with microtubule assemblies, leading to mitotic arrest and subsequent cell death (Anchoori *et al.*, 2008 ▸). Moreover, several N-substituted phenyl­thio­ureas inhibit tyrosinase-mediated melanogenesis and have shown promising activity against melanoma and other hyperpigmentation disorders (Thanigaimalai *et al.*, 2011 ▸).
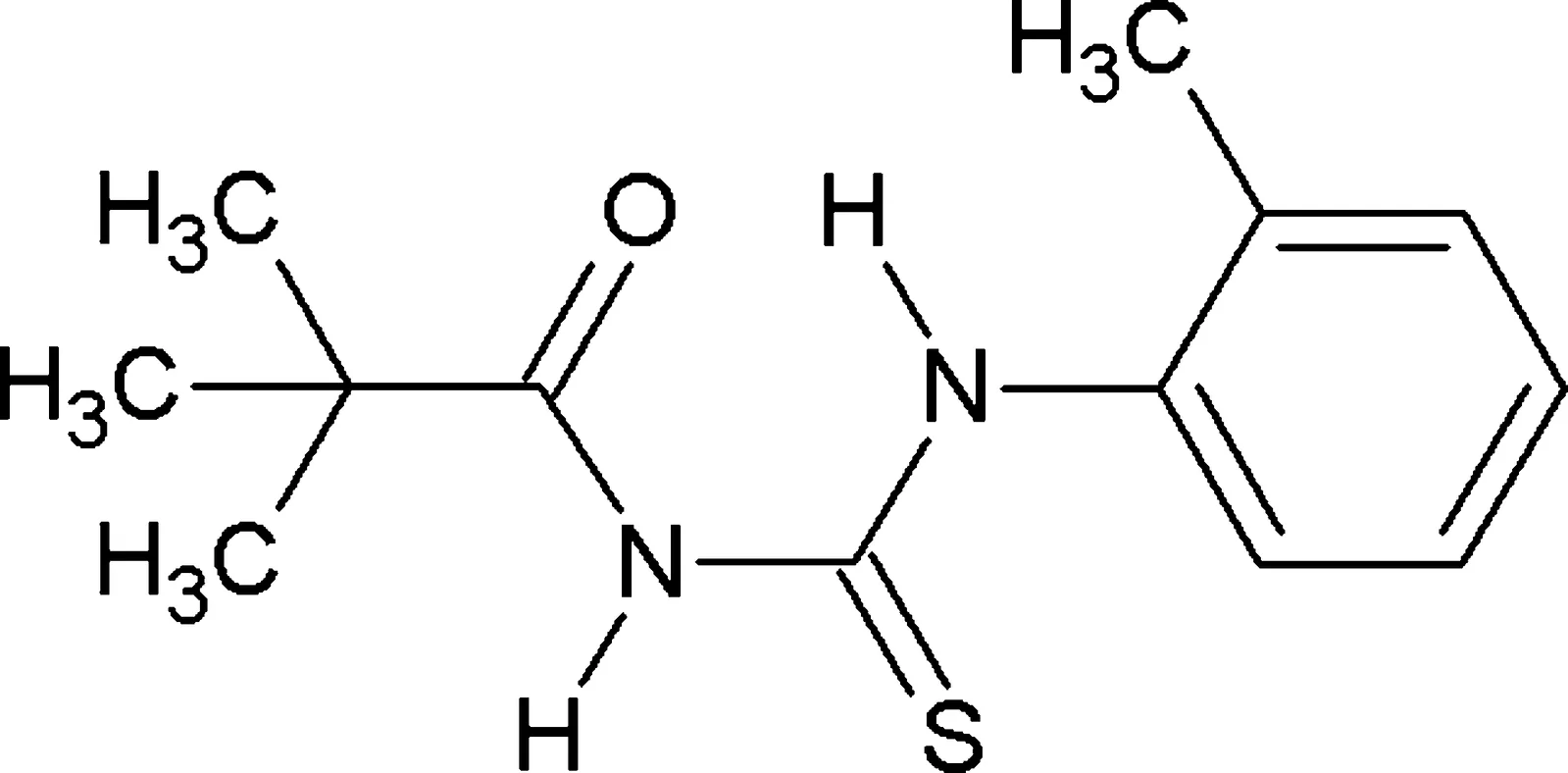


In the context given above, we studied the mol­ecular and crystal structures of the title compound (**I**) and also carried out Hirshfeld surface (HS) and crystal void analyses and calculations of its inter­action energy and energy frameworks.

## 2. Structural commentary

**Figure 1:**
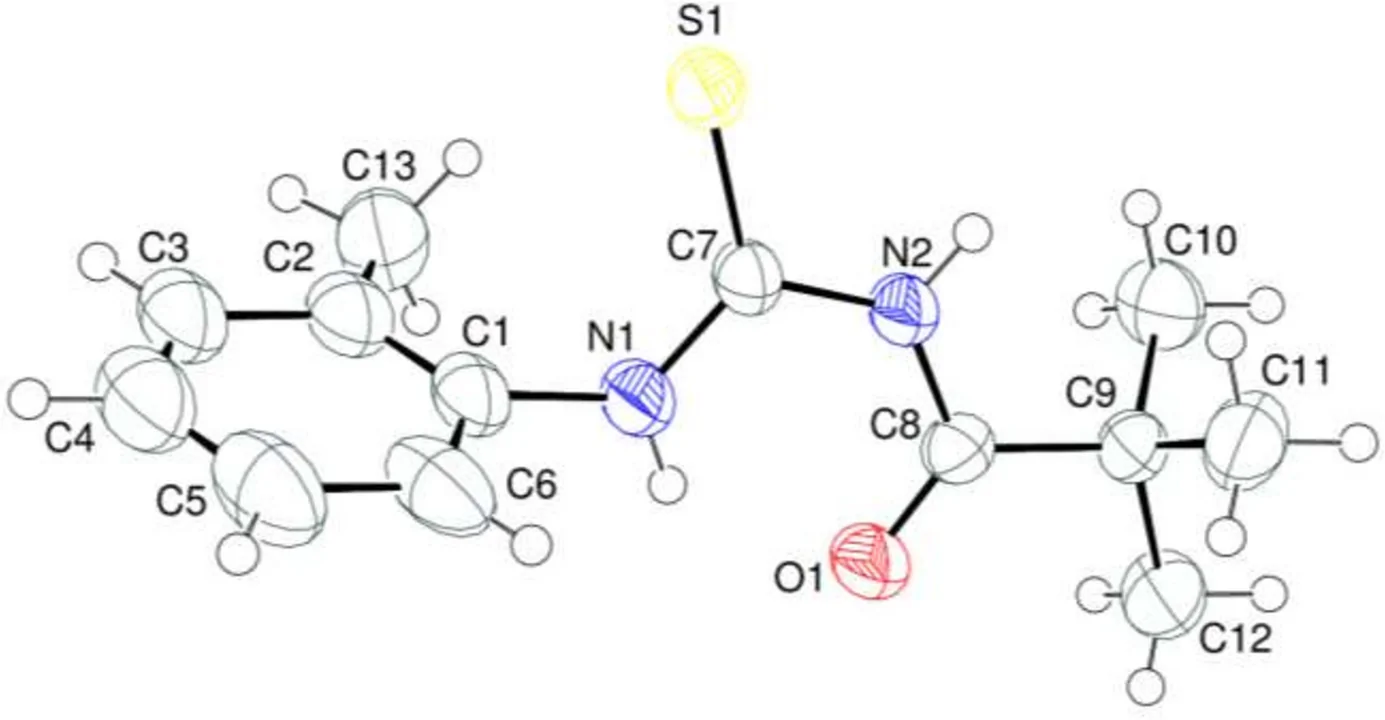
The mol­ecular structure of (**I**) with displacement ellipsoids drawn at the 50% probability level.

**Figure 2:**
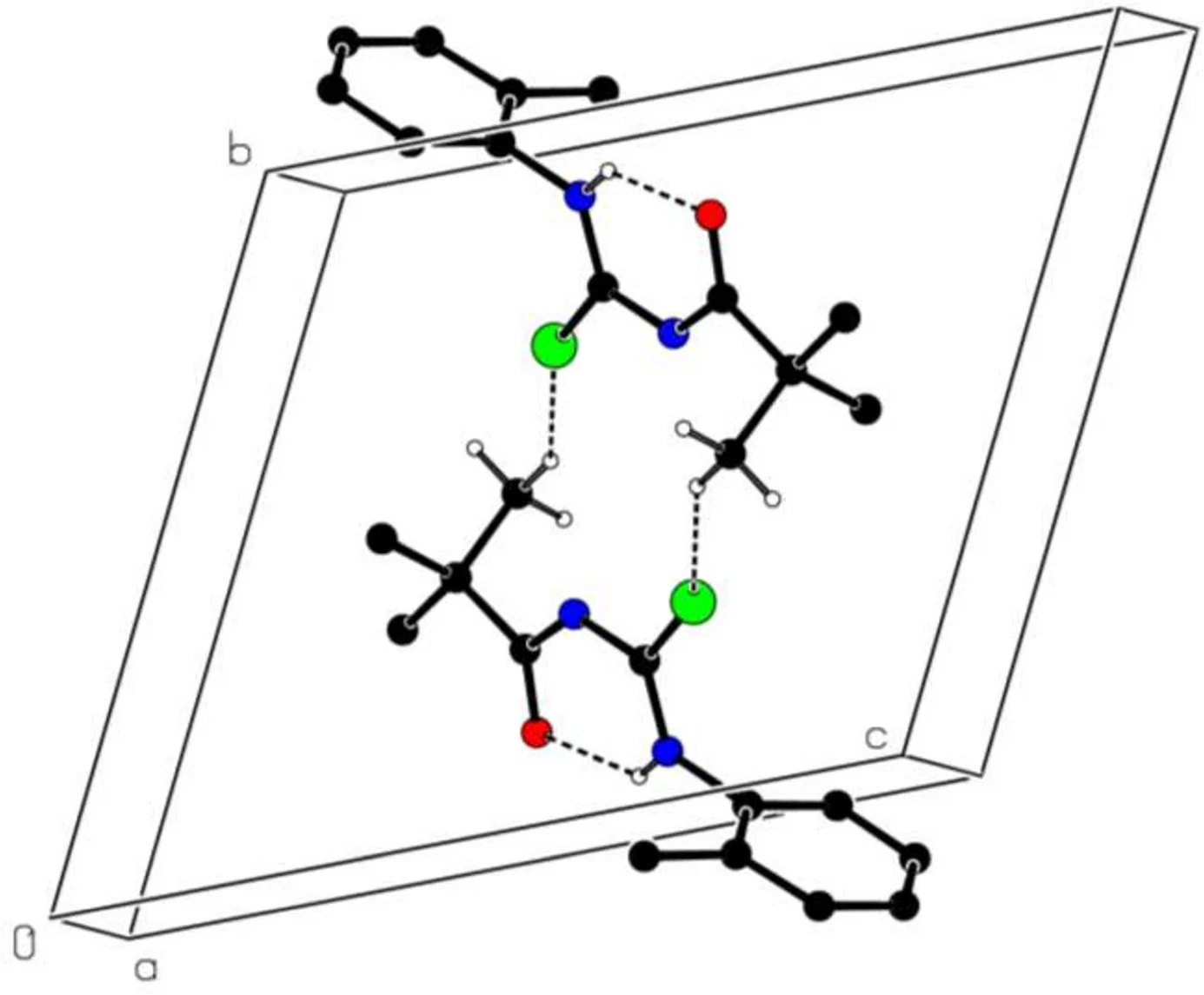
A partial packing diagram of (**I**) viewed down the *a* axis showing the intra­molecular N—H⋯O [*S*(6) ring motif] and inter­molecular C—H⋯S hydrogen bonds [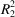
(14) ring motif] as dashed lines.

Compound (**I**) consists of methyl­phenyl and pivaloyl moieties attached to the N atoms of the central thio­urea group (Fig. 1 ▸). An intra­molecular N—H⋯O hydrogen bond (Table 1 ▸) with an *S*(6) ring motif (Etter *et al.*, 1990 ▸) stabilizes the mol­ecular conformation (Fig. 2 ▸).

The planar (O1/C8/C9/C12) and thio­urea (S1/C7/N1/N2) fragments [root-mean-square deviations of 0.017 (3) and 0.002 (2) Å, respectively] subtend a dihedral angle of 3.9 (1)°. The O1, C8, C9 and C12 atoms are 0.049 (2), −0.004 (3), −0.079 (3) and 0.037 (4) Å, respectively, away from the least-squares plane of the thio­urea group. The dihedral angles between the phenyl (C1–C6) ring and the (O1/C8/C9/C12) and thio­urea groups are 81.88 (13) and 78.08 (10)°, respectively. The C13 and N1 atoms are 0.061 (5) and 0.008 (3) Å away from the least-squares plane of the phenyl ring.

The bond lengths (Allen *et al.*, 1987 ▸) and angles in the entire mol­ecule are in normal ranges and comparable to those in similar compounds; for a detailed comparison, see Section 4.

## 3. Supra­molecular features

In the crystal, weak C—H⋯S hydrogen bonds (Table 1 ▸) link the mol­ecules, enclosing 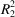
(14) ring motifs (Etter *et al.*, 1990 ▸), into centrosymmetric dimers (Fig. 2 ▸). Furthermore, C—H⋯π(ring) inter­actions (Table 1 ▸) and weak π–π stacking inter­actions between parallel phenyl rings, with centroid-to-centroid distance of 4.134 (2) Å [dihedral angle α = 0.0 (2)° and slippage = 2.288 Å], help to consolidate the packing.

**Figure 3:**
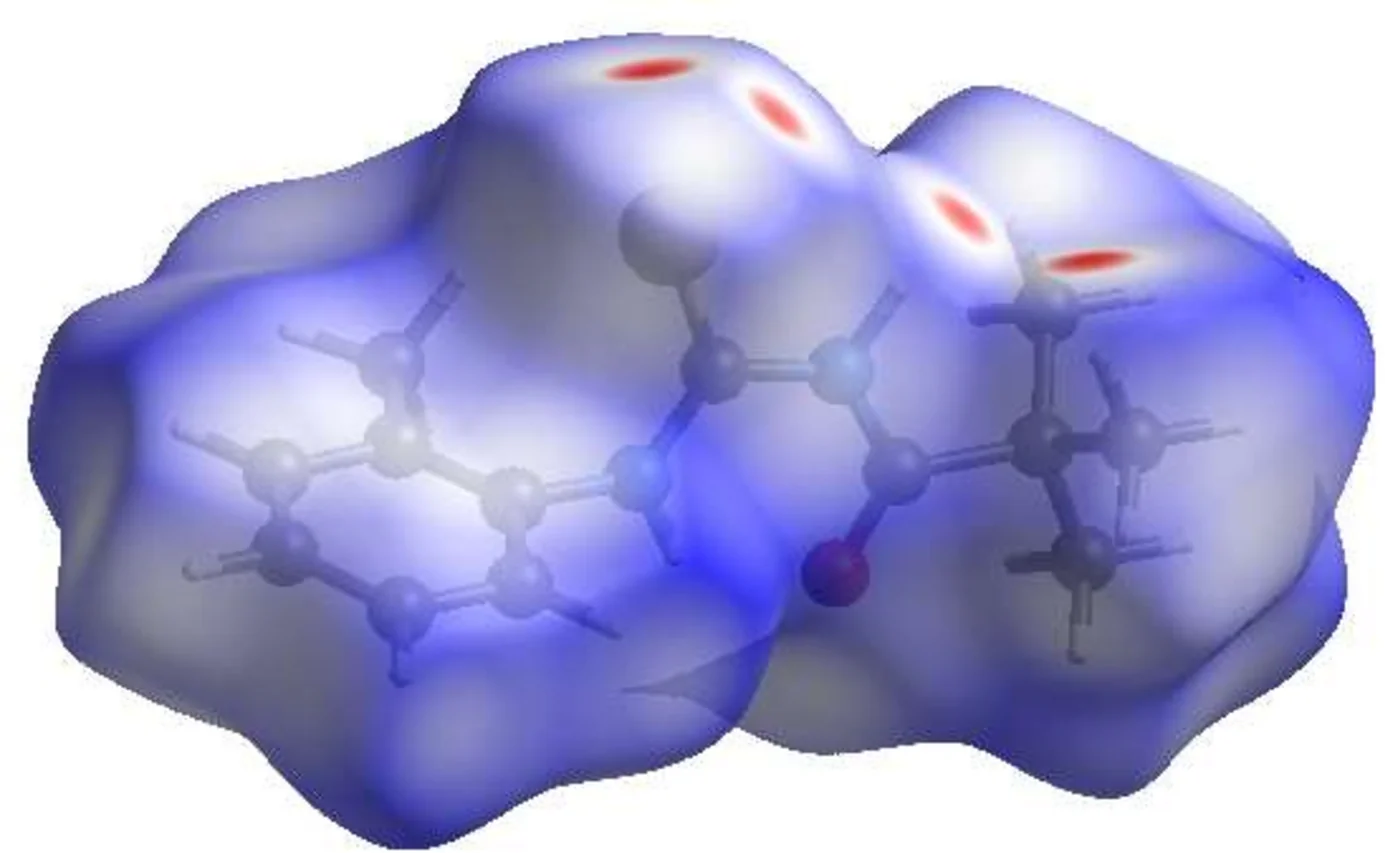
View of the three-dimensional Hirshfeld surface plotted over *d*_norm_.

**Figure 4:**
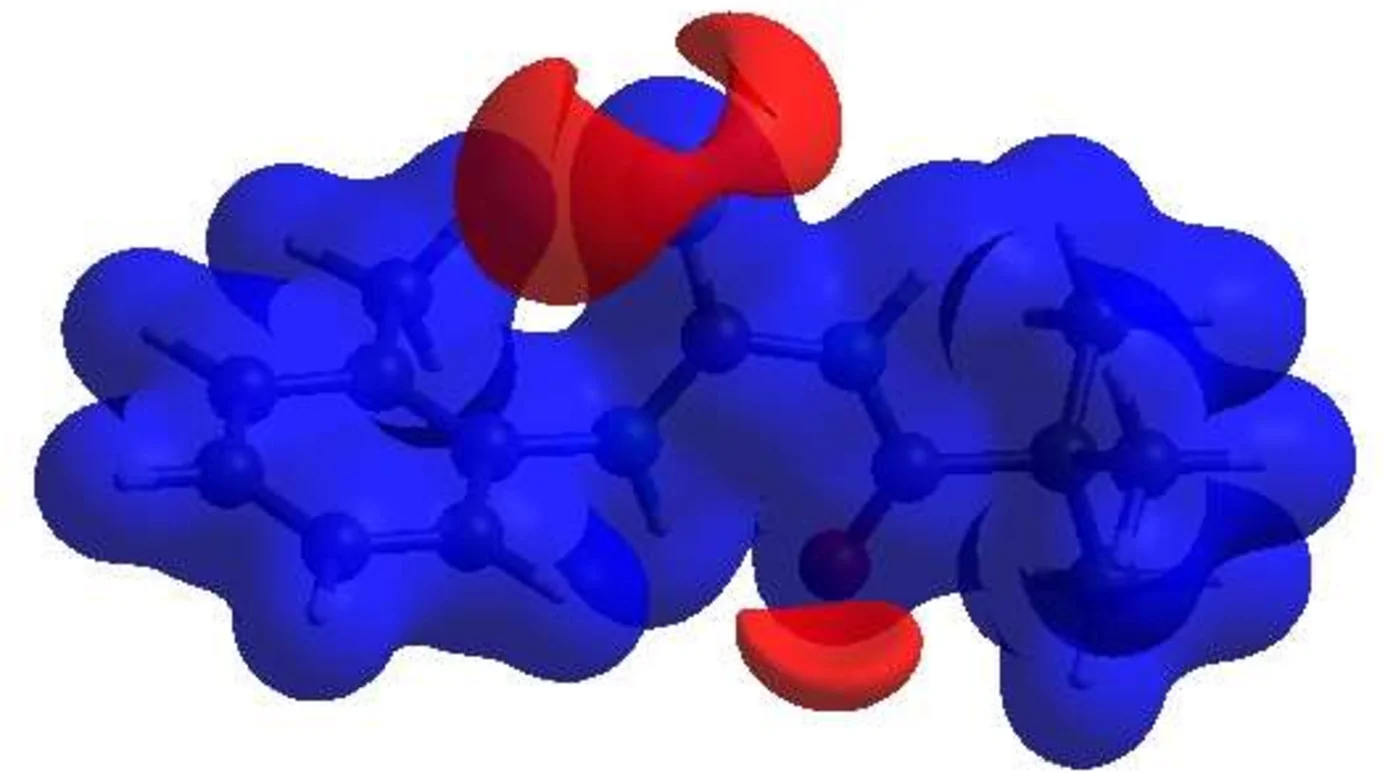
View of the three-dimensional Hirshfeld surface of (**I**) plotted over electrostatic potential using the STO-3 G basis set at the Hartree–Fock level of theory. Hydrogen-bond donors and acceptors are shown as blue and red regions around the atoms, corresponding to positive and negative potentials, respectively.

**Figure 5:**
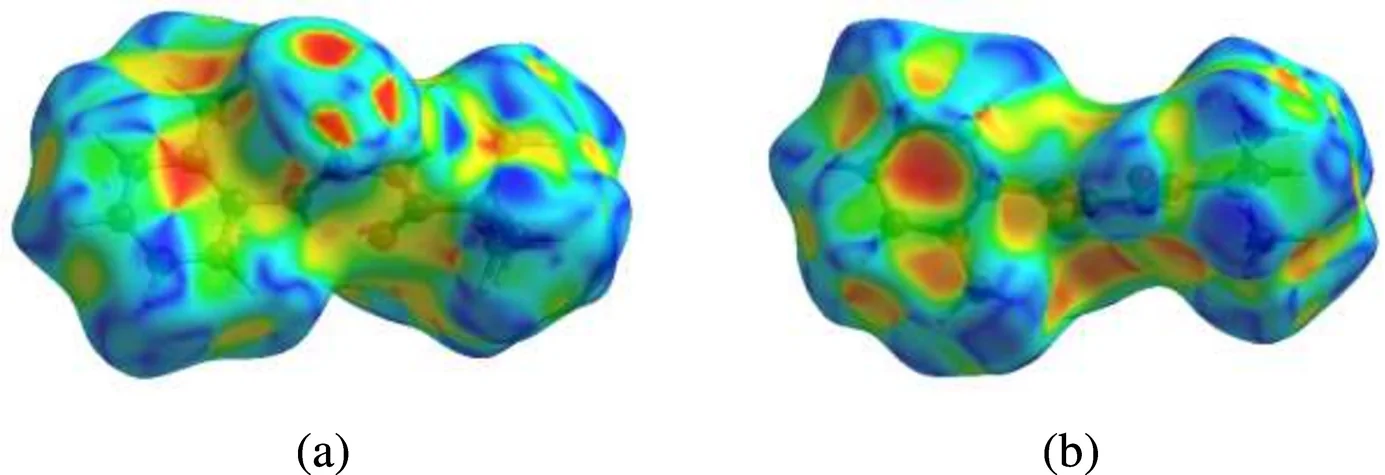
Two orientations of the shape-index, showing (*a*) the π–π and (*b*) the C—H⋯π(ring) inter­actions.

The inter­molecular inter­actions in the crystal were qu­anti­fied by a Hirshfeld surface (HS) analysis using *CrystalExplorer* (Spackman *et al.*, 2021 ▸). Fig. 3 ▸ shows the HS mapped over *d*_norm_ where the white surface indicates contacts with distances equal to the sum of van der Waals radii, and the red and blue colours indicate distances shorter (in close contact) or longer (distinct contacts) than the van der Waals radii, respectively. The red spots indicate their roles as the respective donors and/or acceptors atoms in hydrogen-bonding inter­action, as discussed; they also appear as blue and red regions corresponding to positive and negative potentials on the HS mapped over electrostatic potential (Fig. 4 ▸), indicating positive (hydrogen-bond donors) and negative (hydrogen-bond acceptors) electrostatic potentials. The π–π stacking and the C—H⋯π(ring) inter­actions are indicated in Fig. 5 ▸*a* by the presence of adjacent red and blue triangles, and in Fig. 5 ▸*b* by the presence of red π-holes.

**Figure 6:**
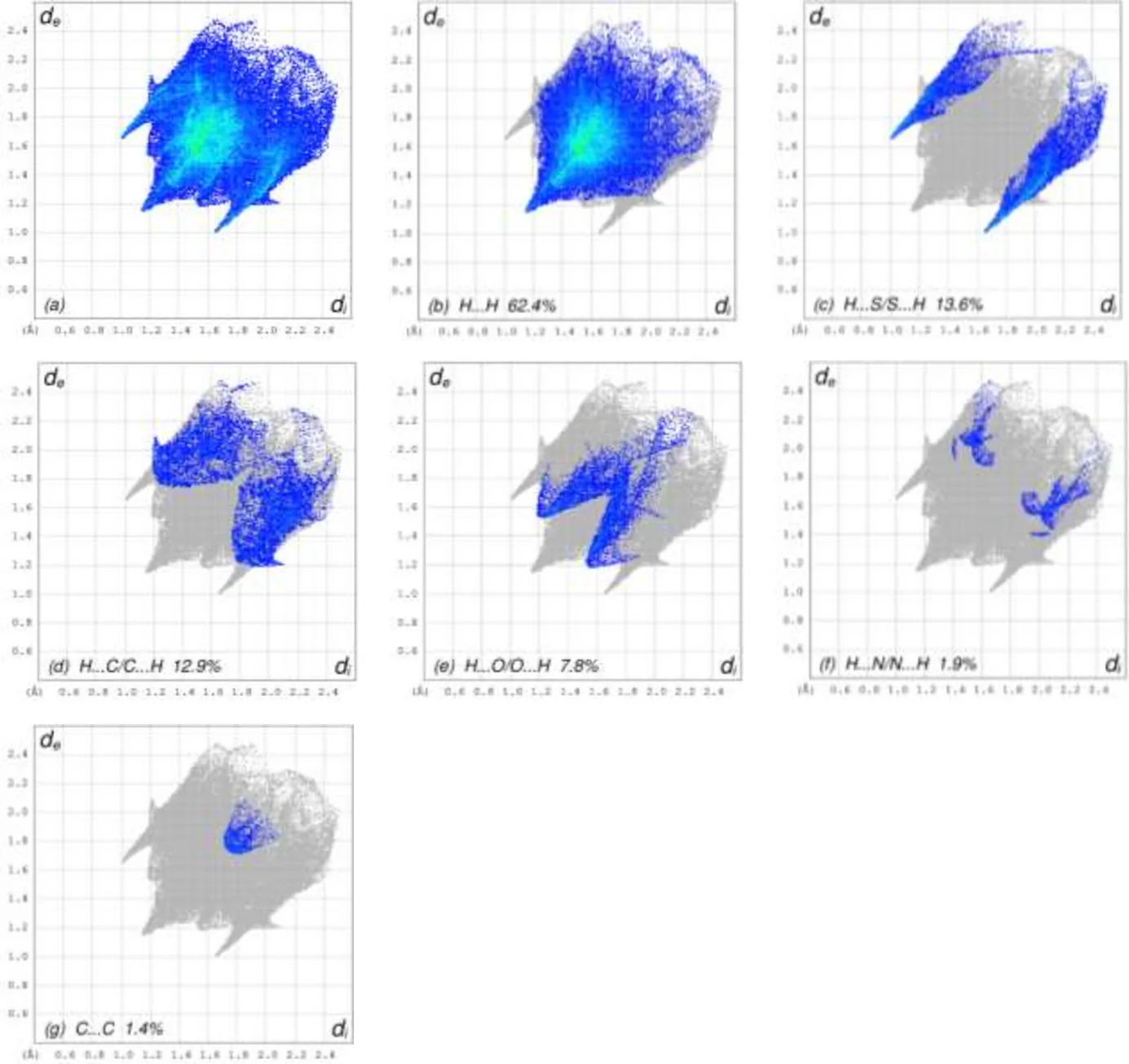
The full two-dimensional fingerprint plots of (**I**), showing (*a*) all inter­actions, and delineated into (*b*) H⋯H, (*c*) H⋯S/S⋯H, (*d*) H⋯C/C⋯H, (*e*) H⋯O/O⋯H, (*f*) H⋯N/N⋯H and (*g*) C⋯C inter­actions. The *d*_i_ and *d*_e_ values are the closest inter­nal and external distances (in Å) from given points on the Hirshfeld surface.

The overall two-dimensional fingerprint plot is shown in Fig. 6 ▸*a*, and those delineated into H⋯H, H⋯S/S⋯H, H⋯C/C⋯H, H⋯O/O⋯H, H⋯N/N⋯H and C⋯C inter­actions are illustrated in Fig. 6 ▸*b–g*, respectively, revealing that H⋯H, H⋯S/S⋯H and H⋯C/C⋯H contacts make the most significant contributions to the HS.

**Figure 7:**
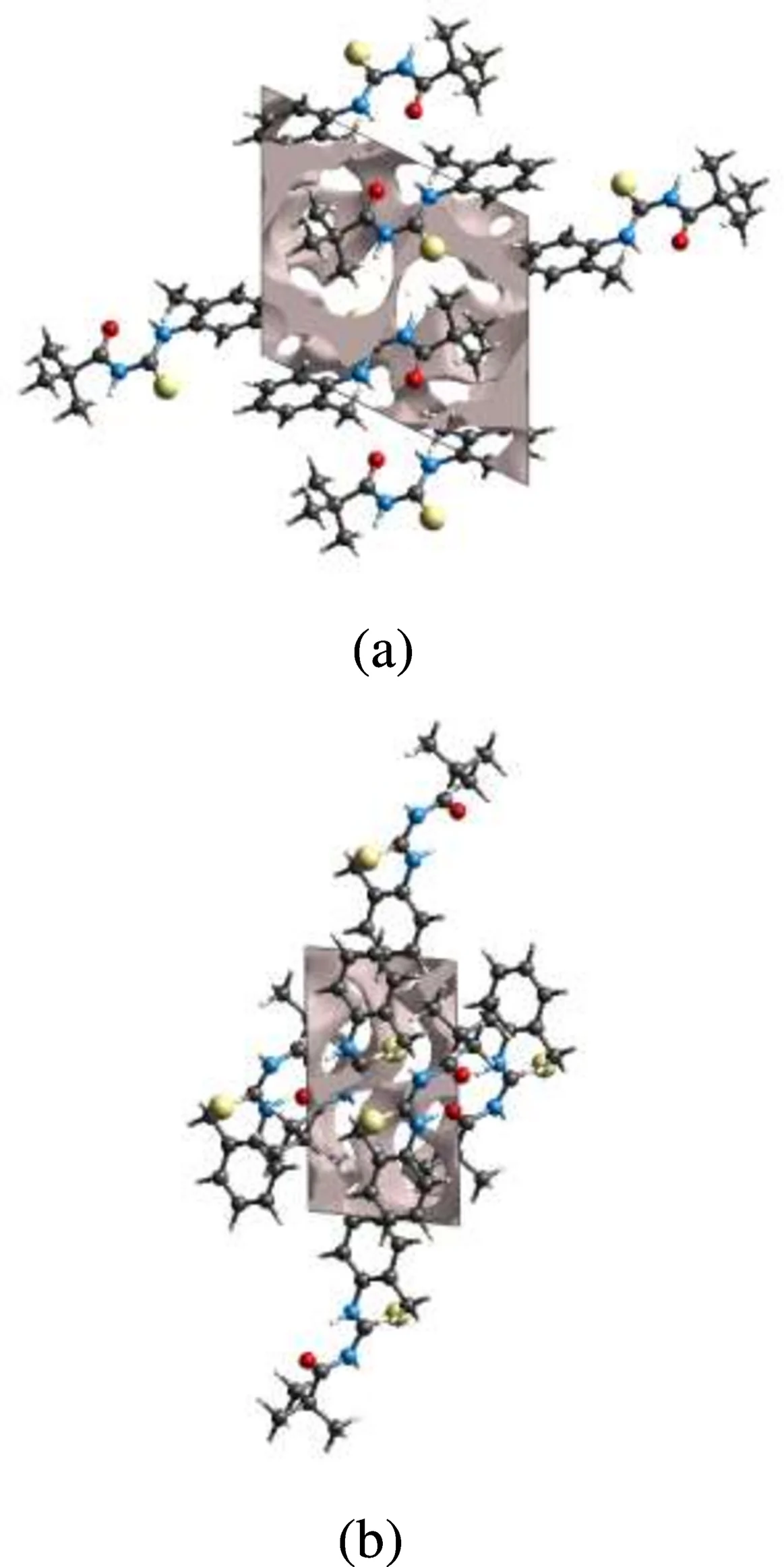
Crystal voids viewed down the (*a*) *a* axis and (*b*) *b* axis.

A void analysis of the crystal packing (Fig. 7 ▸) revealed the void volume and the percentage of free space in the unit cell to be 91.57 Å^3^ and 13.35%, respectively, which indicates a rather compact packing of mol­ecules in the crystal.

Inter­molecular inter­action energies were calculated using the CE–B3LYP/6–31G(d,p) energy model available in *CrystalExplorer* (Spackman *et al.*, 2021 ▸), where a cluster of mol­ecules is generated by applying crystallographic symmetry operations with respect to a selected central mol­ecule within the radius of 3.8 Å by default. The total inter­molecular energy (*E*_tot_) is the sum of electrostatic (*E*_ele_), polarization (*E*_pol_), dispersion (*E*_dis_) and exchange-repulsion (*E*_rep_) energies (Turner *et al.*, 2015 ▸) with scale factors of 1.057, 0.740, 0.871 and 0.618, respectively (Mackenzie *et al.*, 2017 ▸). Hydrogen-bonding inter­action energies (in kJ mol^−1^) were calculated to be −0.6 (*E*_ele_), −0.3 (*E*_pol_), −8.7 (*E*_dis_), 4.9 (*E*_rep_) and −5.4 (*E*_tot_) for the C11—H11*A*⋯S1 hydrogen-bonding inter­action.

**Figure 8:**
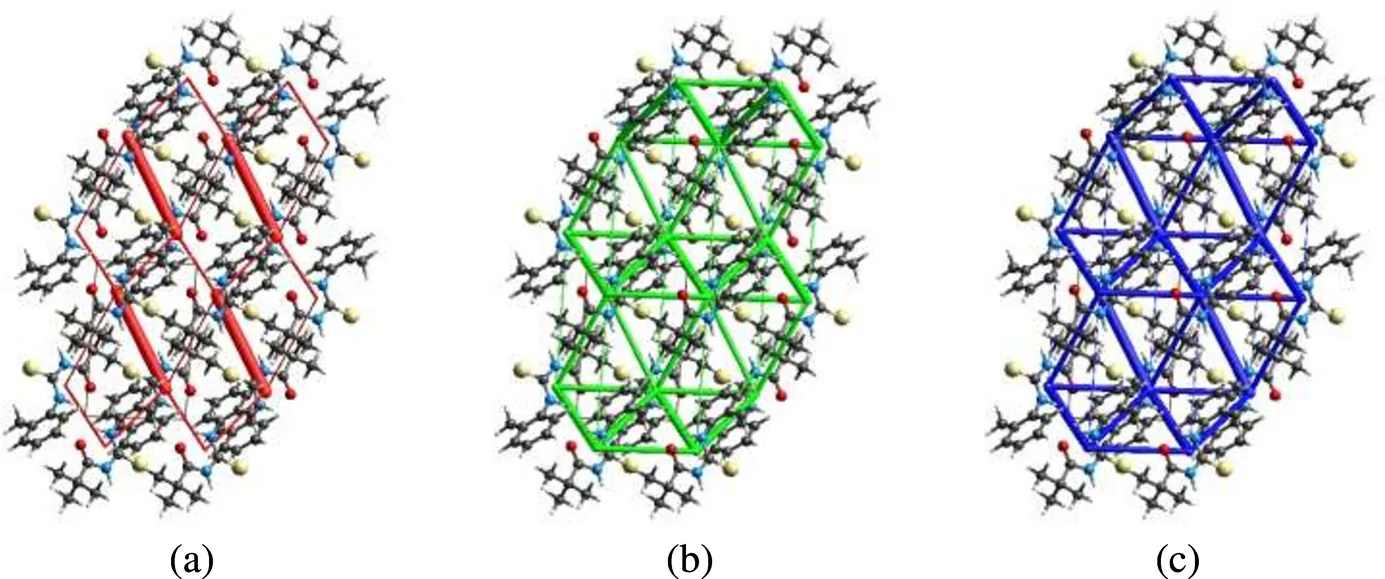
The energy frameworks for a cluster of mol­ecules viewed down the *c* axis showing the (*a*) electrostatic energy, (*b*) dispersion energy and (*c*) total energy diagrams. The cylindrical radius is proportional to the relative strength of the corresponding energies and they were adjusted to the same scale factor of 80 with cut-off value of 5 kJ mol^−1^ within 2×2×2 unit cells.

Energy frameworks combine the calculation of inter­molecular inter­action energies with a graphical representation of their magnitudes, in which they were constructed for *E*_ele_ (red cylinders), *E*_dis_ (green cylinders) and *E*_tot_ (blue cylinders), as shown in Fig. 8 ▸*a*, *b* and *c*. The evaluation of the electrostatic, dispersion and total energy frameworks indicates that the stabilization in the crystal structure is dominated *via* the dispersion energy contributions.

## 4. Database survey

A search of the Cambridge Structural Database (CSD, Version 6.00, updated May 2025; Groom *et al.*, 2016 ▸) revealed six structures closely related to *N*-(2-methyl­phen­yl)-*N*′-pivaloyl­thio­urea. These include 1-(3-nitro­phen­yl)-3-pivaloyl­thio­urea, C_12_H_15_N_3_O_3_S [CSD refcode RIZCUZ; (**II**) Yusof *et al.*, 2008 ▸], 1-(2-nitro­phen­yl)-3-pivaloyl­thio­urea, C_12_H_15_N_3_O_3_S [GINDEN; (**III**) Saeed & Flörke, 2007*a* ▸], 1-(2,4-di­chloro­phen­yl)-3-pivaloyl­thio­urea, C_12_H_14_Cl_2_N_2_OS [HIRDOC; (**IV**) Saeed & Flörke, 2007*b* ▸], 1-(3-bromo­phen­yl)-3-pivaloyl­thio­urea, C_12_H_15_BrN_2_OS [CICREM; (**V**) Sultana *et al.*, 2007*a* ▸], 1-(4-nitro­phen­yl)-3-pivaloyl­thio­urea, C_12_H_15_N_3_O_3_S [GIHFOT; (**VI**) Sultana *et al.*, 2007*b* ▸] and 1-(3-chloro­phen­yl)-3-pivaloyl­thio­urea, C_12_H_15_ClN_2_OS [WIHBAR; (**VII**) Shoukat *et al.*, 2007 ▸],

In the title compound (**I**), the C2—C1—C6 bond angle [122.8 (3)°] is wider than the corresponding ones in compounds (**II**)–(**VI**), and the N1—C7—N2 bond angle (116.4 (2)°) is also wider than those in compounds (**IV** – **VI**). On the other hand, the C1—N1—C7 bond angle [123.3 (2)°) is narrowed with respect to those in compounds (**III**), (**IV**) and (**VI**), and the S1—C7—N1 bond angle [124.4 (2)]) is also narrowed relative to those in compounds (**IV**) and (**VI**).

## 5. Synthesis and crystallization

Compound (**I**) was synthesized by adding a solution of pivaloyl chloride (0.10 mol) in acetone (30 ml) dropwise to a suspension of ammonium thio­cyanate (0.10 mol) in acetone (30 ml). The resulting reaction mixture was stirred for 2 h, and then refluxed for 30 min. After cooling to room temperature, a solution of 2-methyl­aniline (0.10 mol) in acetone (10 ml) was added, and the reaction mixture was refluxed for 3 h. The progress of the reaction was monitored by thin-layer chromatography (TLC). Upon completion, the reaction mixture was poured into cold acidified water (5%_wt_ HCl). The precipitated solid was collected by vacuum filtration, washed thoroughly with water, and dried. White solid, yield 82%, m.p. 371–373 K. IR (KBr) cm^−1^: 3347 (N—H str.), 1179 (C=S str.) and 1681 (C=O str.). ^1^H NMR (400 MHz, CDCl_3_-*d*_6_): δ (ppm) 12.03 (*s*, 1H), 8.50 (*s*, 1H), 7.25–7.62 (*m*, 4H, Ar-H 2-methyl­phenyl ring), 2.34 (*s*, 3H, CH_3_), 1.30 [*s*, 9H, (CH_3_)_3_], ^13^C NMR (400 MHz, CDCl_3_-*d*_6_): δ (ppm) 27.1, 21.0, 134.8, 124.3, 129.4, 136.9, 129.4, 121.3, 171.0, 178.4. Colourless crystals suitable for single-crystal X-ray diffraction analysis were obtained by slow evaporation of an aceto­nitrile solution at room temperature.

## 6. Refinement

Crystal data, data collection and structure refinement details are summarized in Table 2 ▸. Three reflections were omitted from the final refinement cycles, and similarity restraints applied to some of the C atoms of the phenyl ring. Hydrogen atoms attached to the N atoms of thio­urea were located from difference-Fourier maps and refined with a distance restraint of N—H = 0.86 (2) Å, and with *U*_iso_(H) = 1.2*U*_eq_(N). The C-bound hydrogen-atom positions were calculated geometrically at distances of 0.93 Å (aromatic) and 0.96 Å (meth­yl) and refined using a riding model.

## Supplementary Material

Crystal structure: contains datablock(s) I, global. DOI: 10.1107/S205698902600890X/wm5810sup1.cif

Structure factors: contains datablock(s) I. DOI: 10.1107/S205698902600890X/wm5810Isup2.hkl

Supporting information file. DOI: 10.1107/S205698902600890X/wm5810Isup3.cml

CCDC reference: 1816816

Additional supporting information:  crystallographic information; 3D view; checkCIF report

## Figures and Tables

**Table 1:** Hydrogen-bond geometry (Å, °) *Cg*1 is the centroid of the C1–C6 ring.

*D*—H⋯*A*	*D*—H	H⋯*A*	*D*⋯*A*	*D*—H⋯*A*
N1—H1*N*⋯O1	0.84 (2)	1.98 (3)	2.644 (3)	135 (3)
C11—H11*A*⋯S1^i^	0.96	2.79	3.663 (4)	152
C12—H12*C*⋯*Cg*1^ii^	0.96	2.92	3.640 (5)	133

Symmetry codes: (i) 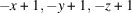; (ii) 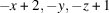.

**Table 2:** Experimental details

Crystal data	
Chemical formula	C_13_H_18_N_2_OS
*M*_r_	250.35
Crystal system, space group	Triclinic, *P* 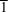
Temperature (K)	293
*a*, *b*, *c* (Å)	6.1050 (4), 10.7020 (3), 11.8910 (3)
α, β, γ (°)	63.290 (4), 81.600 (3), 84.340 (4)
*V* (Å^3^)	686.08 (6)
*Z*	2
Radiation type	Mo *K*α
μ (mm^−1^)	0.22
Crystal size (mm)	0.50 × 0.48 × 0.16

Data collection	
Diffractometer	Oxford Diffraction Xcalibur with Sapphire CCD Detector
Absorption correction	Multi-scan (*CrysAlis RED*; Oxford Diffraction, 2009 ▸)
*T*_min_, *T*_max_	0.897, 0.965
No. of measured, independent and observed [*I* > 2σ(*I*)] reflections	4335, 2756, 2287
*R*_int_	0.010
(sin θ/λ)_max_ (Å^−1^)	0.625

Refinement	
*R*[*F*^2^ > 2σ(*F*^2^)], *wR*(*F*^2^), *S*	0.064, 0.164, 1.16
No. of reflections	2756
No. of parameters	160
No. of restraints	3
H-atom treatment	H atoms treated by a mixture of independent and constrained refinement
Δρ_max_, Δρ_min_ (e Å^−3^)	0.71, −0.24

Computer programs: *CrysAlis CCD* and *CrysAlis RED* (Oxford Diffraction, 2009 ▸), *SHELXT* (Sheldrick, 2015*a* ▸), *SHELXL* (Sheldrick, 2015*b* ▸) and *ORTEP-3 for Windows* and *WinGX* (Farrugia, 2012 ▸).

## supplementary crystallographic information

### 3-(2,2-Dimethylpropanoyl)-1-(2-methylphenyl)thiourea Crystal data

**Table:**

C_13_H_18_N_2_OS	*Z* = 2
*M**_r_* = 250.35	*F*(000) = 268
Triclinic, *P*1	*D*_x_ = 1.212 Mg m^−^^3^
*a* = 6.1050 (4) Å	Mo *K*α radiation, λ = 0.71073 Å
*b* = 10.7020 (3) Å	Cell parameters from 2155 reflections
*c* = 11.8910 (3) Å	θ = 3.4–27.7°
α = 63.290 (4)°	µ = 0.22 mm^−^^1^
β = 81.600 (3)°	*T* = 293 K
γ = 84.340 (4)°	Prism, colourless
*V* = 686.08 (6) Å^3^	0.50 × 0.48 × 0.16 mm

### 3-(2,2-Dimethylpropanoyl)-1-(2-methylphenyl)thiourea Data collection

**Table:**

Oxford Diffraction Xcalibur with Sapphire CCD Detector diffractometer	2287 reflections with *I* > 2σ(*I*)
Rotation method data acquisition using ω scans.	*R*_int_ = 0.010
Absorption correction: multi-scan (*CrysAlis RED*; Oxford Diffraction, 2009)	θ_max_ = 26.4°, θ_min_ = 3.4°
*T*_min_ = 0.897, *T*_max_ = 0.965	*h* = −7→7
4335 measured reflections	*k* = −13→13
2756 independent reflections	*l* = −10→14

### 3-(2,2-Dimethylpropanoyl)-1-(2-methylphenyl)thiourea Refinement

**Table:**

Refinement on *F*^2^	3 restraints
Least-squares matrix: full	Hydrogen site location: mixed
*R*[*F*^2^ > 2σ(*F*^2^)] = 0.064	H atoms treated by a mixture of independent and constrained refinement
*wR*(*F*^2^) = 0.164	*w* = 1/[σ^2^(*F*_o_^2^) + (0.0561*P*)^2^ + 0.6536*P*] where *P* = (*F*_o_^2^ + 2*F*_c_^2^)/3
*S* = 1.16	(Δ/σ)_max_ < 0.001
2756 reflections	Δρ_max_ = 0.71 e Å^−^^3^
160 parameters	Δρ_min_ = −0.24 e Å^−^^3^

### 3-(2,2-Dimethylpropanoyl)-1-(2-methylphenyl)thiourea Special details

**Table:**

Geometry. All esds (except the esd in the dihedral angle between two l.s. planes) are estimated using the full covariance matrix. The cell esds are taken into account individually in the estimation of esds in distances, angles and torsion angles; correlations between esds in cell parameters are only used when they are defined by crystal symmetry. An approximate (isotropic) treatment of cell esds is used for estimating esds involving l.s. planes.

### 3-(2,2-Dimethylpropanoyl)-1-(2-methylphenyl)thiourea Fractional atomic coordinates and isotropic or equivalent isotropic displacement parameters (Å^2^)

**Table:**

	*x*	*y*	*z*	*U*_iso_*/*U*_eq_	
C1	0.6535 (6)	0.0041 (3)	0.7571 (3)	0.0553 (8)	
C2	0.4729 (6)	−0.0708 (3)	0.7789 (3)	0.0582 (8)	
C3	0.4096 (7)	−0.1702 (4)	0.9074 (3)	0.0694 (10)	
H3	0.285478	−0.223253	0.927043	0.083*	
C4	0.5310 (8)	−0.1862 (4)	0.9995 (4)	0.0765 (11)	
H4	0.486587	−0.250364	1.082242	0.092*	
C5	0.7150 (8)	−0.1130 (4)	0.9772 (3)	0.0817 (12)	
H5	0.795826	−0.128953	1.043172	0.098*	
C6	0.7811 (7)	−0.0141 (4)	0.8549 (3)	0.0712 (10)	
H6	0.904989	0.038526	0.837313	0.085*	
C7	0.6455 (4)	0.2367 (3)	0.5778 (2)	0.0404 (6)	
C8	0.9416 (4)	0.3001 (3)	0.3944 (2)	0.0407 (6)	
C9	1.0284 (5)	0.4254 (3)	0.2737 (3)	0.0441 (6)	
C10	0.8441 (6)	0.4912 (4)	0.1868 (3)	0.0629 (9)	
H10A	0.722387	0.522879	0.229562	0.094*	
H10B	0.900447	0.569074	0.110502	0.094*	
H10C	0.794361	0.422614	0.165898	0.094*	
C11	1.1069 (6)	0.5315 (4)	0.3110 (4)	0.0657 (9)	
H11A	0.984175	0.562679	0.353691	0.099*	
H11B	1.219945	0.488307	0.366534	0.099*	
H11C	1.165792	0.610173	0.236291	0.099*	
C12	1.2214 (6)	0.3751 (4)	0.2060 (4)	0.0735 (11)	
H12A	1.277779	0.453031	0.129752	0.110*	
H12B	1.336465	0.334007	0.260380	0.110*	
H12C	1.171636	0.306594	0.185095	0.110*	
C13	0.3526 (7)	−0.0550 (4)	0.6784 (4)	0.0762 (10)	
H13A	0.230783	−0.116751	0.712468	0.114*	
H13B	0.297175	0.039974	0.636992	0.114*	
H13C	0.449045	−0.077743	0.618279	0.114*	
N1	0.7287 (4)	0.1062 (2)	0.6302 (2)	0.0503 (6)	
N2	0.7550 (4)	0.3273 (2)	0.4621 (2)	0.0441 (6)	
O1	1.0292 (3)	0.1835 (2)	0.4296 (2)	0.0567 (6)	
S1	0.42204 (13)	0.29558 (7)	0.64054 (7)	0.0537 (3)	
H1N	0.841 (4)	0.084 (4)	0.592 (3)	0.064*	
H2N	0.703 (6)	0.411 (2)	0.434 (3)	0.064*	

### 3-(2,2-Dimethylpropanoyl)-1-(2-methylphenyl)thiourea Atomic displacement parameters (Å^2^)

**Table:**

	*U*^11^	*U*^22^	*U*^33^	*U*^12^	*U*^13^	*U*^23^
C1	0.066 (2)	0.0377 (14)	0.0473 (16)	0.0103 (13)	0.0110 (14)	−0.0129 (12)
C2	0.066 (2)	0.0470 (16)	0.0556 (18)	0.0094 (15)	0.0014 (15)	−0.0217 (14)
C3	0.084 (3)	0.0492 (18)	0.057 (2)	0.0141 (17)	0.0096 (18)	−0.0166 (16)
C4	0.094 (3)	0.062 (2)	0.055 (2)	0.016 (2)	0.007 (2)	−0.0182 (17)
C5	0.108 (3)	0.074 (2)	0.0485 (17)	0.021 (2)	−0.0178 (19)	−0.0174 (15)
C6	0.093 (3)	0.061 (2)	0.0482 (16)	0.0275 (18)	−0.0149 (17)	−0.0186 (13)
C7	0.0446 (14)	0.0353 (13)	0.0340 (13)	0.0004 (11)	0.0000 (11)	−0.0106 (10)
C8	0.0368 (13)	0.0431 (14)	0.0385 (14)	−0.0004 (11)	−0.0010 (11)	−0.0161 (11)
C9	0.0429 (14)	0.0396 (14)	0.0414 (14)	−0.0011 (11)	0.0065 (11)	−0.0139 (11)
C10	0.072 (2)	0.064 (2)	0.0398 (16)	0.0038 (17)	−0.0042 (15)	−0.0138 (15)
C11	0.059 (2)	0.061 (2)	0.076 (2)	−0.0176 (16)	0.0085 (17)	−0.0317 (18)
C12	0.069 (2)	0.061 (2)	0.066 (2)	0.0026 (17)	0.0296 (17)	−0.0180 (17)
C13	0.082 (3)	0.065 (2)	0.074 (2)	−0.0051 (19)	−0.004 (2)	−0.0240 (19)
N1	0.0527 (14)	0.0404 (12)	0.0407 (13)	0.0072 (11)	0.0092 (11)	−0.0088 (10)
N2	0.0442 (12)	0.0354 (11)	0.0394 (12)	0.0033 (9)	0.0058 (10)	−0.0088 (10)
O1	0.0537 (12)	0.0438 (11)	0.0547 (12)	0.0094 (9)	0.0079 (10)	−0.0123 (9)
S1	0.0573 (5)	0.0395 (4)	0.0482 (4)	0.0048 (3)	0.0144 (3)	−0.0124 (3)

### 3-(2,2-Dimethylpropanoyl)-1-(2-methylphenyl)thiourea Geometric parameters (Å, º)

**Table:**

C1—C2	1.353 (5)	C9—C12	1.521 (4)
C1—C6	1.424 (5)	C9—C11	1.529 (4)
C1—N1	1.448 (4)	C9—C10	1.536 (4)
C2—C13	1.430 (5)	C10—H10A	0.9600
C2—C3	1.436 (5)	C10—H10B	0.9600
C3—C4	1.350 (6)	C10—H10C	0.9600
C3—H3	0.9300	C11—H11A	0.9600
C4—C5	1.358 (6)	C11—H11B	0.9600
C4—H4	0.9300	C11—H11C	0.9600
C5—C6	1.388 (5)	C12—H12A	0.9600
C5—H5	0.9300	C12—H12B	0.9600
C6—H6	0.9300	C12—H12C	0.9600
C7—N1	1.329 (3)	C13—H13A	0.9600
C7—N2	1.393 (3)	C13—H13B	0.9600
C7—S1	1.665 (3)	C13—H13C	0.9600
C8—O1	1.218 (3)	N1—H1N	0.840 (18)
C8—N2	1.381 (3)	N2—H2N	0.852 (18)
C8—C9	1.525 (4)		
			
C2—C1—C6	122.8 (3)	C9—C10—H10A	109.5
C2—C1—N1	120.5 (3)	C9—C10—H10B	109.5
C6—C1—N1	116.7 (3)	H10A—C10—H10B	109.5
C1—C2—C13	121.8 (3)	C9—C10—H10C	109.5
C1—C2—C3	117.1 (3)	H10A—C10—H10C	109.5
C13—C2—C3	121.1 (4)	H10B—C10—H10C	109.5
C4—C3—C2	119.7 (4)	C9—C11—H11A	109.5
C4—C3—H3	120.2	C9—C11—H11B	109.5
C2—C3—H3	120.2	H11A—C11—H11B	109.5
C3—C4—C5	123.2 (4)	C9—C11—H11C	109.5
C3—C4—H4	118.4	H11A—C11—H11C	109.5
C5—C4—H4	118.4	H11B—C11—H11C	109.5
C4—C5—C6	119.4 (4)	C9—C12—H12A	109.5
C4—C5—H5	120.3	C9—C12—H12B	109.5
C6—C5—H5	120.3	H12A—C12—H12B	109.5
C5—C6—C1	117.9 (4)	C9—C12—H12C	109.5
C5—C6—H6	121.0	H12A—C12—H12C	109.5
C1—C6—H6	121.0	H12B—C12—H12C	109.5
N1—C7—N2	116.4 (2)	C2—C13—H13A	109.5
N1—C7—S1	124.4 (2)	C2—C13—H13B	109.5
N2—C7—S1	119.26 (19)	H13A—C13—H13B	109.5
O1—C8—N2	121.7 (2)	C2—C13—H13C	109.5
O1—C8—C9	122.8 (2)	H13A—C13—H13C	109.5
N2—C8—C9	115.5 (2)	H13B—C13—H13C	109.5
C12—C9—C8	108.6 (2)	C7—N1—C1	123.3 (2)
C12—C9—C11	110.0 (3)	C7—N1—H1N	119 (2)
C8—C9—C11	108.3 (2)	C1—N1—H1N	117 (2)
C12—C9—C10	109.5 (3)	C8—N2—C7	128.5 (2)
C8—C9—C10	109.9 (2)	C8—N2—H2N	118 (2)
C11—C9—C10	110.5 (3)	C7—N2—H2N	114 (2)
			
C6—C1—C2—C13	−177.3 (3)	O1—C8—C9—C11	−115.0 (3)
N1—C1—C2—C13	1.3 (5)	N2—C8—C9—C11	65.0 (3)
C6—C1—C2—C3	1.2 (4)	O1—C8—C9—C10	124.2 (3)
N1—C1—C2—C3	179.8 (3)	N2—C8—C9—C10	−55.8 (3)
C1—C2—C3—C4	−0.8 (5)	N2—C7—N1—C1	171.6 (3)
C13—C2—C3—C4	177.7 (3)	S1—C7—N1—C1	−9.1 (5)
C2—C3—C4—C5	−0.6 (6)	C2—C1—N1—C7	84.0 (4)
C3—C4—C5—C6	1.6 (6)	C6—C1—N1—C7	−97.4 (4)
C4—C5—C6—C1	−1.1 (5)	O1—C8—N2—C7	3.5 (5)
C2—C1—C6—C5	−0.3 (5)	C9—C8—N2—C7	−176.5 (3)
N1—C1—C6—C5	−178.9 (3)	N1—C7—N2—C8	−0.8 (4)
O1—C8—C9—C12	4.4 (4)	S1—C7—N2—C8	179.9 (2)
N2—C8—C9—C12	−175.6 (3)		

### 3-(2,2-Dimethylpropanoyl)-1-(2-methylphenyl)thiourea Hydrogen-bond geometry (Å, º)

*Cg*1 is the centroid of the C1–C6 ring.

**Table:**

*D*—H···*A*	*D*—H	H···*A*	*D*···*A*	*D*—H···*A*
N1—H1*N*···O1	0.84 (2)	1.98 (3)	2.644 (3)	135 (3)
C11—H11*A*···S1^i^	0.96	2.79	3.663 (4)	152
C12—H12*C*···Cg1^ii^	0.96	2.92	3.640 (5)	133

Symmetry codes: (i) −*x*+1, −*y*+1, −*z*+1; (ii) −*x*+2, −*y*, −*z*+1.
